# Generation and ex vivo characterization of a full‐thickness substitute of the human urethra by tissue engineering

**DOI:** 10.1002/btm2.70049

**Published:** 2025-09-16

**Authors:** David Sánchez‐Porras, Miguel Etayo‐Escanilla, José‐Andrés Moreno‐Delgado, María del Mar Lozano‐Martí, Fabiola Bermejo‐Casares, Miguel Alaminos, Jesús Chato‐Astrain, Fernando Campos, M. Carmen Sánchez‐Quevedo, Ricardo Fernández‐Valadés

**Affiliations:** ^1^ Department of Histology, Tissue Engineering Group, School of Medicine University of Granada Granada Spain; ^2^ Instituto de Investigación Biosanitaria ibs.GRANADA Granada Spain; ^3^ Division of Pediatric Surgery University Hospital Virgen de Las Nieves Granada Spain

**Keywords:** endothelium, fibrin‐agarose biomaterials, human urethra, smooth muscle, spongy layer, tissue engineering, tunica albuginea, urethral mucosa

## Abstract

Tissue engineering may offer efficient alternatives for the surgical repair of severe conditions affecting the human urethra. However, development of tubular full‐thickness substitutes is challenging. In this work, we have generated and evaluated ex vivo a novel full‐thickness human urethra substitute (FHUS) containing its three main layers: the urethral mucosa (UM), the spongy layer (SP), and the tunica albuginea (AL). Results first showed that the generation of a FHUS significantly improved the biomechanical properties of this artificial tissue as compared to the individual layers, although the resistance of the native urethra was not reached. At the structural level, we found that FHUS shared important histological similarities with the native urethra. Analysis of the individual layers showed that UM had a stratified epithelium that expressed several epithelial markers, including cytokeratins CK7 and CK14, uroplakin 1b, and the intercellular junction proteins desmoplakin, tight junction protein 1, and claudin. At the stromal level, UM tended to increase the presence of collagen fibers and versican with time. The SP layer displayed abundant CD31 and CD34‐positive blood vessels, but small amounts of collagen and proteoglycans. The AL layer showed scattered smooth muscle cells expressing α‐smooth muscle actin, smoothelin, and desmin cell markers, and contained low amounts of collagen and proteoglycans. Analysis of the basement membrane components collagen IV and laminin revealed their progressive development with time, especially collagen IV. These results confirm the possibility of developing a partially biomimetic full‐thickness substitute of human urethra that might have potential clinical usefulness for the clinical repair of severe urethral lesions.

**Translational Impact Statement**

This is the first report in which a full‐thickness biomimetic human urethral tubular substitute (including the urethral mucosa and the spongy and albuginea layers) has been generated by tissue engineering. The characterization performed on each individual layer used to generate this full‐thickness substitute confirms the possibility of generating in vitro a viable urethral substitute with optimal mechanical properties and expression of the main native markers, being the first step toward the future clinical translation of this artificial organ in patients with severe conditions affecting the native urethra.

## 1 INTRODUCTION

The human urethra is a complex tubular organ mainly consisting of a urethral mucosa (UM) in direct contact with the lumen of the urethra, a spongy layer (SP), and an external tunica albuginea layer (AL) surrounding the SP,^1^ especially in males, although some of these structures are less developed in women.^2^ In turn, the UM is formed by a stratified non‐cornified squamous epithelium with an underlying stromal layer.^3^ This multilayered structure can be damaged for different reasons, leading to malfunctioning of the human urethra.

Although serious conditions of the urethra are not very prevalent, diseases that affect this structure are very difficult to manage.^4^ In particular, most structural diseases affecting the urethra in pediatric patients are related to congenital malformations, such as hypospadias and epispadias,^5^ whereas urethral diseases in adult patients are often associated with urethral strictures and urethral defects associated with trauma, iatrogenic causes, infections, and tumors.^6^ In most cases, current treatment is based on the surgical correction of the urethral defect in a procedure called urethroplasty, which is often dependent on the use of tissue flaps or autografts that are not always available.^7^ Unfortunately, results of this surgical procedure are still suboptimal, especially in cases with large urethral defects,^7^ and novel therapeutic approaches able to improve these results are in need.

In this sense, the generation of human bioartificial tissues and organs by tissue engineering has previously proven to be potentially useful for the clinical treatment of severe diseases affecting the different organs of the human body.^8^, ^9^ Based on the use of human cells combined with biomaterials and growth factors, bioartificial tissues generated by tissue engineering should be biocompatible and able to biomimetically reproduce the structure and composition of the native tissues.^10^ In this regard, we recently showed that the use of highly biocompatible fibrin‐agarose biomaterials allowed the efficient biomimetic generation of bioengineered substitutes of the human skin^11^ and cornea^12^ that demonstrated clinical usefulness in patients with severe skin burns and corneal ulcers, respectively.

In the field of the human urethra, several tissue engineered tissues have been described to date,^13^ although very few of them have been clinically tested, including some acellular tissue substitutes based on collagen biomaterials,^14^ and cellular oral mucosa substitutes generated by tissue engineering.^15^, ^16^ Most bioengineered urethral substitutes make use of a single type of cells combined with different types of biomaterials. However, it has been demonstrated that the more efficient models of bioartificial urethra were obtained when several types of cells were co‐cultured.^17^

Despite current biofabrication methods allowing the efficient generation of single‐layer bioartificial tissues, a completely biomimetic substitute for the human urethra, including all three layers of this organ, has not yet been reported. In this milieu, the application of plastic compression nanostructuration techniques has demonstrated the ability to generate multilayered substitutes of the hard palate, as previously reported.^18^ However, this technique has not been applied to generate a multilayered substitute of the human urethra.

In the present work, we generated a substitute of each individual layer of the human urethra (UM, SP, and AL layers) using fibrin‐agarose biomaterials with different types of urethral cells, and we have carried out a comprehensive characterization analysis of each layer to determine its degree of biomimicry with the human native urethra. Then, we have fabricated a novel full‐thickness human urethra substitute (FHUS) containing the three layers by using nanostructuration methods.

## 2 METHODS

### 2.1 Tissue samples, cell cultures, and generation of urethral substitutes

Primary cultures of normal human cells corresponding to different tissues were purchased from Innoprot (Derio, Spain), including bladder stromal fibroblasts (P10988), urothelial cells (P10952), smooth muscle cells or leiomyocytes (P10951), and microvascular cells (P10989). Cells were cultured using standard culture conditions at 37°C with 5% CO_2_, using the culture media and protocols indicated by the supplier for each type of cell. Cells were trypsinized at subconfluence using a mixture of trypsin and Ethylenediaminetetraacetic Acid (EDTA) (Merck, Darmstadt, Germany) and expanded in new culture flasks.

To generate a bioengineered substitute of each of the three main layers of the human urethra, including the UM layer, SP, and tunica AL layer, each cell type was combined with fibrin (SP) or fibrin‐agarose hydrogels (UM and AL), following previously described biofabrication protocols.^19^, ^20^ In all cases, a cellular stromal substitute was first fabricated, and in the case of the UM, which in the human urethra consists of a stratified epithelial layer and a subjacent stromal layer, an epithelium was subsequently cultured on top. Briefly, to generate 500 μL of hydrogel, 380 μL of human plasma were mixed with 62.5 μL of culture medium containing the specific cell type of each tissue layer (10^5^ cultured fibroblasts for the UM, 10^5^ microvascular cells for the SP and 2 × 10^5^ leiomyocytes for the AL), 7.5 μL of tranexamic acid (Amchafibrin™, Fides‐Ecofarma, Spain), and 25 μL of 2% calcium chloride (Merck). In the case of the tissues based on fibrin‐agarose hydrogels (UM and AL), 25 μL of a 2% type‐VII agarose (Merck) solution melted in Phosphate‐Buffered Saline (PBS) (Merck) were added to the mixture at the last step of the procedure, whereas 25 μL of PBS were added to generate the SP substitutes. This mixture was aliquoted in 12‐well culture plates with porous culture inserts (Sarstedt, Nümbrecht, Germany) and allowed to jellify for 24 h in a cell incubator at 37°C. In the case of the UM substitute, 7.5 × 10^4^ urothelial cells were then subcultured on top of the stromal substitute, and the air‐liquid culture technique was applied to induce epithelial stratification and differentiation, as previously reported.^21^ In all cases, each bioengineered tissue layer was cultured using its respective culture medium and kept for 14 days at 37°C in a cell incubator.

Once the three main layers of the human urethra (UM, SP, and AL) were generated by tissue engineering, we constructed a FHUS containing these three layers using nanostructuration methods, as previously reported.^18^ In brief, the AL substitute was carefully placed on top of a sterile nylon membrane with a pore diameter of 0.22 μm (GE Healthcare, Chicago, IL, USA) positioned on several layers of 3MM filter paper (Thermo Fisher Scientific, Waltham, MA, USA). Then, the SP substitute was placed on top, followed by the UM layer (with the epithelial layer on top), forming a plywood‐like structure. Afterwards, a nylon surgical mesh was used to cover the epithelium, followed by a second nylon membrane with a pore diameter of 0.22 μm and several layers of sterile 3MM filter paper. Then, 500 g of weight was applied for 3 min to induce plastic compression nanostructuration and fusion of the three tissue layers to form a three‐layered substitute that was sutured around a silicon tutor tube to form a hollow tubular structure able to reproduce the three‐dimensional form of the human native urethra that was considered as a FHUS. In this study, eight different tubular FHUS constructs (*n* = 8) were generated, and their diameter and thickness were subsequently measured.

As controls, small fragments of the normal human urethra were obtained from male donors accepting their participation in the study. In short, small pieces of urethral tissue attached to urological organs excised from patients with urological conditions (benign prostate or bladder diseases) were obtained and processed for histological analysis as described below. None of the samples were removed from the patient specifically for the study, and all samples corresponded to tissues discarded after the previously scheduled surgical procedure.

### 2.2 Cell viability

To assess the viability of cells included in each layer of the urethral substitute (UM, SP, and AL) corresponding to each follow‐up time (2, 7, and 14 days), we used the Live/Dead (LD) cell viability assay (Invitrogen, Waltham, MA, USA), following the manufacturer's instructions adapted to three‐dimensional structures. For this, three different samples were analyzed per time and per experimental group (*n* = 3). Tissue samples corresponding to UM, SP, and AL layers were nanostructured and incubated in a PBS solution containing 0.05% of calcein AM and 0.2% of ethidium homodimer‐1 for 10 min. Then, samples were washed in PBS, placed on a glass slide, and carefully coverslipped. This resulted in a thin, optically accessible layer that allowed full‐depth analysis with an Eclipse i90 fluorescence microscope (Nikon, Tokyo, Japan) using green and red fluorescence, as previously reported.^22^, ^23^ Cell survival was quantified across the entire thickness of the constructs for each tissue type and time point. For quantification, three alternate fields were randomly selected and analyzed in each sample. The percentage of live and dead cells in each tissue type and each follow‐up time was calculated using FIJI software v2.14.0/1.54f (NIH, Bethesda, MD, USA).

### 2.3 Histology, histochemistry, and immunohistochemistry

Tissue samples corresponding to the different bioartificial tissues generated in this work at each analysis time, and control normal human urethra were fixed for 24 h in buffered formalin (Panreac Química S.L.U., Barcelona, Spain), dehydrated using a graded series of ethanol, cleared in xylene, and embedded in paraffin following routine histological analysis protocols. Histological sections of 2 μm thickness were then obtained, mounted on glass slides, deparaffinized, and rehydrated. For a general structural analysis, sections were stained with hematoxylin and eosin (HE) following standard staining methods, and images were obtained using a Pannoramic DESK II DW scanner (3D Histotech, Budapest, Hungary).

To determine the proliferation rate of the epithelial cells corresponding to UM tissues, we carried out indirect immunohistochemistry analyses for the cell proliferation marker KI‐67, and the percentage of proliferative cells showing positive expression of this marker was determined in each experimental group. To evaluate epithelial differentiation, UM tissues were subjected to immunohistochemistry for cytokeratins 7 and 14 (CK7 and CK14) and uroplakin 1b (UPK1b), whereas the intercellular junction proteins desmoplakin (DSP), tight junction protein 1 (TJP1) and claudin were evaluated by immunofluorescence. Indirect immunohistochemistry was also used to assess the presence of two relevant components of the basement membrane (collagen IV and laminin) and a key component of the extracellular matrix (ECM) (versican, VCAN) in UM, SP, and AL. The presence of blood vessels was revealed in SP using the vascular differentiation markers CD31 and CD34,^24^ whereas leiomyocytes were labeled using α‐smooth muscle actin (SMA‐ACT), smoothelin (SMOO) and desmin markers.^25^ Technical details related to the immunohistochemical procedures (antibodies, pretreatments, and references) are summarized in Table S1.

In addition, fibrillar collagens of the tissue ECM were stained using picrosirius red (PSR) histochemistry. For this, samples were incubated in F3B sirius red reagent for 30 min, followed by Harris's hematoxylin counterstaining for 5 min. Identification of non‐fibrillar ECM proteoglycans was carried out using alcian blue (AB) histochemical methods by incubating the tissue sections in AB working solution for 30 min and counterstained with nuclear fast red for 1 min. In both cases, stained samples were dehydrated and coverslipped.

For scanning electron microscopy analysis (SEM), sections of the bioengineered FHUS were fixed in 2.5% buffered glutaraldehyde (Panreac Química S.L.U.), washed three times in 0.05 M cacodylate buffer pH 7.2 (Merck), dehydrated with increasing concentrations of acetone, dried using the critical point method, and sputter coated with gold–palladium. Samples were mounted on aluminum stubs and examined in a FEI Quanta 200 environmental scanning electron microscope (FEI Europe, Eindhoven, Netherlands).

For these histological, histochemical, and immunohistochemical analyses, eight different samples (*n* = 8) were analyzed for each type of sample (UM, SP, AL, and FHUS).

### 2.4 Biomechanical analyses

In order to determine the biomechanical properties of the different tissue substitutes generated in this present study, nanostructured UM, SP, AL, and FHUS corresponding to Day 14 of development were subjected to tensile testing using a biomechanical analysis device Instron 5943 (Instron, Norwood, MA, USA). In each case, rectangular tissue sections with an approximate size of 30 × 10 mm were placed on the analysis machine and fixed to the analysis clamps, with a free distance of 10 mm between both clamps. The test was performed by applying a constant tensile stress between both clamps, with a constant strain rate of 5 mm/min, until the sample was fractured. A 50 N load cell was used to analyze the biomechanical behavior of each sample. In each sample, the Instron Blue Hill 2 Material Testing software was used to calculate the Young modulus, corresponding to the tangent modulus of the initial linear section of the stress–strain curve; the stress at fracture break, corresponding to the point on the stress–strain curve where fracture of the sample occurred; and the traction deformation, calculated as the total elongation of the sample before fracture (expressed as percentage).^22^ As controls, tissue sections of the native male porcine urethra with the same size as the bioengineered samples were obtained at a local slaughterhouse, cleaned to remove all rests or adipose tissue or other structures different from the three main layers of the native urethra (UM, SP, and AL), and biomechanically analyzed as described for bioengineered tissues. Eight different samples (*n* = 8) were analyzed for each type of sample (UM, SP, AL, and FHUS), based on previous studies by the research group suggesting that this sample size allows statistical comparisons with high confidence and reproducibility.^26^, ^27^, ^28^, ^29^

### 2.5 Quantification and statistical analysis

First, the immunohistochemical signal was semiquantitatively assessed for the basement membrane markers analyzed in UM, SP, and AL (collagen IV and laminin). For this, three expert histologists analyzed each image and scored the reaction signal as strong (+++), mild (++), slight (+), or negative (−), using a previously reported scale.^30^

The analysis of cell viability in each tissue layer was performed by splitting channels and applying a threshold to green and red channels in LD images per time and urethral layer, whereas the number of KI‐67 positive and negative cells was quantified to determine the cell proliferation profile in 6 images for each condition (*n* = 6).

For the analysis of epithelial markers in the UM layer and stromal components in each cell layer, including ECM molecules, blood vessels, and leiomyocytes, we quantified the staining signal using the ImageJ software (National Institutes of Health, Bethesda, MD, USA), as previously reported.^30^ In brief, images corresponding to the histochemical methods PSR and AB and the immunohistochemical analysis of UPK1b, CK7, CK14, DSP, TJP1, claudin, VCAN, CD31, CD34, SMA‐ACT, SMOO, and desmin were converted to binary—black and white—after selecting the specific color channel for each analysis method, and the percentage of tissue occupied by positive signal (area fraction) was automatically quantified by the ImageJ program after selecting a square area of 50 × 50 μm. Eight measurements were obtained per type of sample (*n* = 8).

Statistical comparisons of the epithelial markers, stromal components (ECM molecules, blood vessels and leiomyocytes) and biomechanical properties results were performed using the Real Statistics Resource Pack software (Release 7.2) (Dr. Charles Zaiontz, Purdue University, West Lafayette, IN, USA), available at https://real-statistics.com. First, the normality of data distribution for each variable was assessed using the Shapiro–Wilk test. Since the majority of variables did not fit a normal distribution, non‐parametric tests were selected for further analyses. Specifically, the Mann‐Whitney *U* test was used for pairwise comparisons between two specific groups. For all quantitative analyses, a sample size of *n* = 8 was used per group. For the stromal components, we performed comparative analyses between the control group (CTR) and the experimental groups at each follow‐up time (Days 2, 7, and 14), along with comparisons between two specific time points to evaluate temporal changes in tissue responses. For the biomechanical properties, statistical comparisons were conducted between the CTR and each individual type of tissue substitute (UM, SP, AL, and FHUS), as well as between specific types of tissue substitutes to evaluate differences in mechanical performance across the different constructs. To control type I error due to multiple comparisons, a Bonferroni correction was applied, and a Bonferroni‐adjusted *p*‐value of 0.001 was set for statistical significance to increase the statistical confidence in the observed results.

## 3 RESULTS

### 3.1 Analysis of cell viability and cell proliferation of each bioengineered urethral layer (UM, SP, and AL) generated by tissue engineering

**FIGURE 1:**
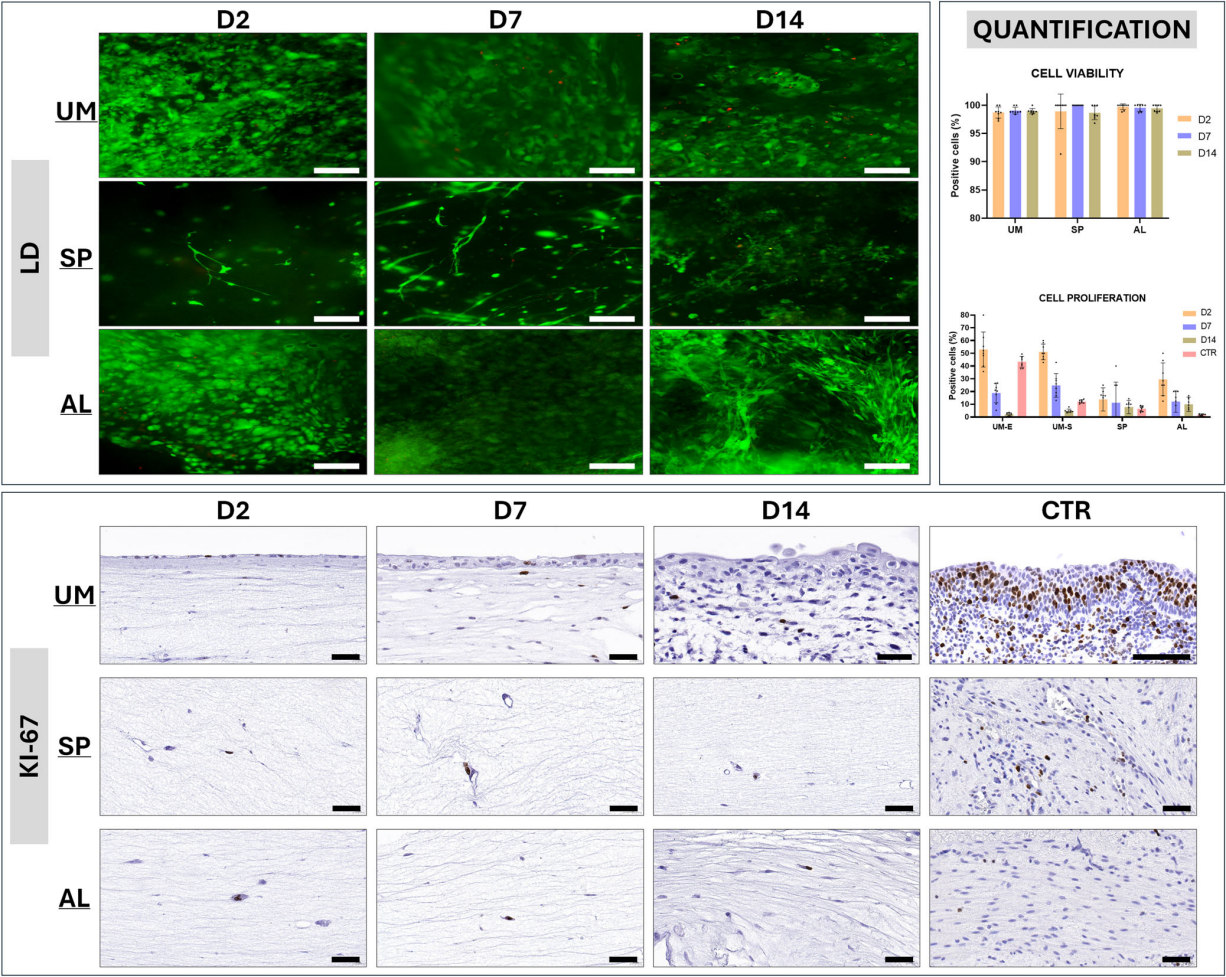
Analysis of cell viability of each urethral layer substitute (urethral mucosa layer [UM], spongy layer [SP], and tunica albuginea layer [AL]) kept in culture for 2, 7, and 14 days (D2, D7, and D14, respectively) using Live/Dead (LD) and immunohistochemistry of each urethral layer substitute (UM, SP, and AL) and human native urethra for the cell proliferation marker KI‐67. Results of the quantitative analysis of the percentage of viable cells and proliferating cells are shown in the histograms (*n* = 8). CTR, human native urethra used as control. Images correspond to illustrative pictures obtained from each type of sample, with a sample size of *n* = 8 per group. Scale bars: 20 μm in LD and 50 μm in KI‐67.

When we analyzed the viability of the cells found in each individual tissue layer (UM, SP, and AL) kept in culture for 2, 7, and 14 days (Figure 1), we found that most cells remained viable, with all samples showing more than 98% of viable cells. Then, our analysis of cell proliferation, as determined by KI‐67 expression, revealed that the highest percentage of proliferating cells corresponded to bioartificial tissues kept ex vivo for 2 days (D2 samples). As shown in Figure 1, the percentage of proliferating cells tended to decrease with culture time. Interestingly, the lowest proliferation levels were found in the SP and AL layers of CTR samples, although the epithelial cells of CTR showed high proliferation rates.

### 3.2 Histological analysis of each bioengineered urethral layer (UM, SP, and AL) generated by tissue engineering and CTR native urethra

**FIGURE 2:**
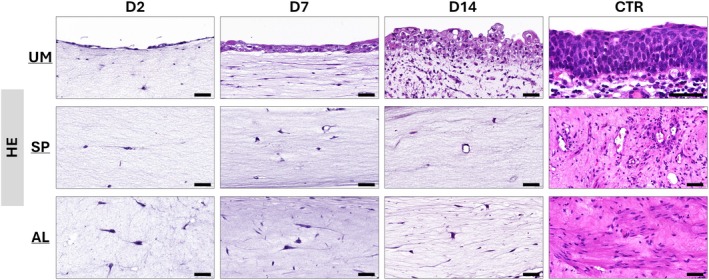
Histological analysis of each urethral layer substitute (urethral mucosa layer [UM], spongy layer [SP], and tunica albuginea layer [AL]) kept in culture for 2, 7, and 14 days (D2, D7, and D14, respectively) and human native urethra using hematoxylin and eosin (HE) staining. Images correspond to illustrative pictures obtained from each type of sample, with a sample size of *n* = 8 per group. CTR, human native urethra used as control. Scale bars: 50 μm.

Histological analysis of each individual layer generated in the laboratory (UM, SP, and AL), along with the human native urethra used as a control, using HE staining (Figure 2) revealed several differences among samples. First, we found that the UM layer consisted of a very thin epithelial layer with only one stratum of cells and a stromal layer containing a regular biomaterial in which scarce cells could be found. When this structure was analyzed at Day 7 of follow‐up, we found a stratified epithelium with two to four cell strata, and cells in the stroma were more abundant and elongated than at the previous analysis time. At Day 14, we found a thicker epithelium with up to 10 cell strata, and the stroma was densely populated by elongated and spindle‐shaped cells. As compared to the native urethra used as a control, the epithelial layer of UM showed lower levels of differentiation, without the fine structure and organization of the strata in the native urethra epithelium, although the number of cells in the stroma was comparable to the UM. Regarding the SP layer, we found that this structure consisted of a regular, dense material in which cells were scattered, with few differences in the number of cells among the three follow‐up times analyzed here. However, we found that cells tended to organize in hollow structures resembling microvasculature at Days 7 and 14 of ex vivo development. In the case of the CTR, these microvascular structures were more abundant, and the number of cells surrounding the microvessels was higher as compared to SP. Finally, the histological analysis of the AL layer revealed the presence of abundant elongated cells from Day 2 to Day 14, with a trend to increase the number of cells at Days 7 and 14 as compared to Day 2. This structure partially resembled that of the CTR tissue, although the native urethra contained a higher number of cells and these cells tended to organize in bundles with abundant cytoplasm that were compatible with the presence of well‐organized muscle tissue, which was absent in the bioengineered tissues.

### 3.3 Histochemical, immunohistochemical, and immunofluorescence analysis of the urethral mucosa layer generated by tissue engineering and CTR tissues

**FIGURE 3:**
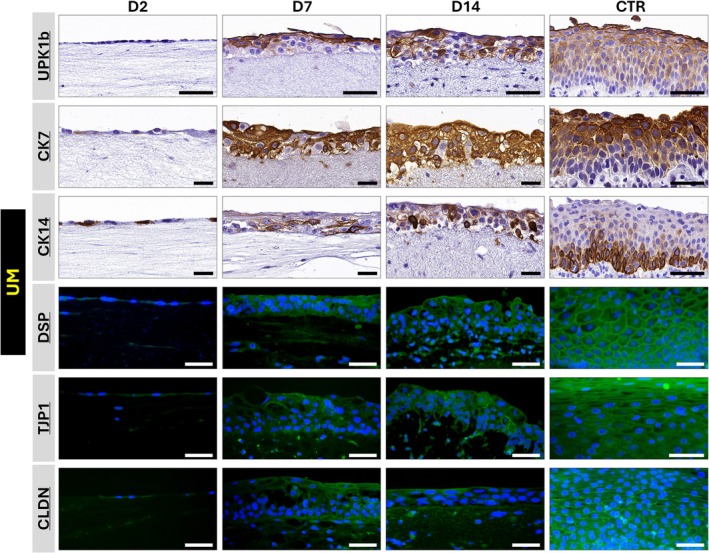
Analysis of epithelial cell markers in the bioengineered urethral mucosa layer (UM) generated by tissue engineering and kept in culture for 2 days (D2), 7 days (D7), and 14 days (D14), and in the native human urethra used as control (CTR) using immunohistochemistry and immunofluorescence. CK7, cytokeratin 7; CK14, cytokeratin 14; CLDN, claudin; DSP, desmoplakin; TJP1, tight junction protein 1; UPK1b, uroplakin 1b. Images correspond to illustrative pictures obtained from each type of sample, with a sample size of *n* = 8 per group. Scale bars: 50 μm.

Analysis of relevant markers of the human UM using histochemical, immunohistochemical, and immunofluorescence methods (Figure 3 and Table 1) revealed that the epithelium of the human native urethra (CTR samples) was strongly positive for all the analyzed epithelial markers, including UPK1b, CK7, CK14, DSP, TJP1, and claudin. In contrast, bioartificial UM tissues initially showed very low or negative expression of all these markers at Day 2 of development, with significant differences versus CTR, and tended to increase at Days 7 and 14, although the highly positive levels found in CTR samples were not reached for any of the analyzed markers, and differences with CTR were statistically significant at Days 7 and 14 for all markers. Interestingly, samples corresponding to Days 7 and 14 were very similar, with statistically significant differences found only for CK14.

**TABLE 1:** Quantitative analysis of epithelial and stromal components, including extracellular matrix molecules, blood vessels and leiomyocytes, in each layer of the human urethra substitute generated by tissue engineering (urethral mucosa layer [UM], spongy layer [SP], and tunica albuginea layer [AL]) and kept in culture for 2 days (D2), 7 days (D7), and 14 days (D14) and in the human native urethra used as control (CTR) (*n* = 8). Top rows show the average and standard deviation results obtained for each type of tissue, whereas lower rows correspond to statistical *p* values for the comparison of two specific samples using the Mann‐Whitney test.

	UM									SP					AL					
	UPK1b	CK7	CK14	DSP	TJP1	CLDN	PSR	AB	VCAN	PSR	AB	VCAN	CD31	CD34	PSR	AB	VCAN	SMA‐ACT	SMOO	DESMIN
D2	10.65 ± 4.47	18.41 ± 5.24	10.76 ± 6.26	9.83 ± 7.11	0.61 ± 0.31	1.81 ± 1.84	0.59 ± 0.47	0.29 ± 0.33	1.15 ± 0.75	0.46 ± 0.48	0.23 ± 0.18	1.39 ± 1.34	1.74 ± 2.23	1.34 ± 1.35	1.87 ± 1.00	0.42 ± 0.37	4.03 ± 1.92	3.98 ± 2.10	2.08 ± 1.36	1.54 ± 1.86
D7	39.37 ± 10.2	69.8 ± 11.56	36.7 ± 1.82	43.05 ± 15.74	19.81 ± 12.66	21.31 ± 11.84	0.72 ± 0.73	0.81 ± 0.58	12.45 ± 3.81	0.53 ± 0.53	0.47 ± 0.49	2.49 ± 2.29	1.31 ± 1.47	1.00 ± 1.31	1.55 ± 1.08	0.31 ± 0.48	18.52 ± 6.03	4.69 ± 2.62	4.75 ± 2.46	3.22 ± 1.76
D14	46.87 ± 8.47	75.83 ± 3.83	56.77 ± 10.15	50.23 ± 17.75	21.47 ± 6.65	25.33 ± 12.31	9.82 ± 3.46	1.26 ± 1.67	29.05 ± 3.32	0.66 ± 0.39	0.45 ± 0.65	4.95 ± 2.48	2.18 ± 2.23	1.70 ± 1.17	3.40 ± 2.36	0.73 ± 0.53	19.54 ± 4.21	6.51 ± 1.98	5.02 ± 1.59	8.40 ± 3.22
CTR	73.26 ± 9.57	91.57 ± 4.47	78.14 ± 7.37	91.6 ± 6.98	79.79 ± 9.26	87.82 ± 14.29	84.55 ± 4.25	17.08 ± 4.18	63.89 ± 10.62	97.24 ± 2.47	3.63 ± 2.37	34.12 ± 7.57	13.04 ± 9.11	16.44 ± 8.86	97.65 ± 2.95	16.50 ± 11.1	50.34 ± 4.85	39.35 ± 11.22	36.4 ± 18.34	39.37 ± 7.30
CTR versus D2	0.0002*	0.0002*	0.0002*	0.0002*	0.0002*	0.0002*	0.0002*	0.0002*	0.0002*	0.0002*	0.0002*	0.0002*	0.0006*	0.0002*	0.0002*	0.0002*	0.0002*	0.0002*	0.0002*	0.0002*
CTR versus D7	0.0002*	0.0002*	0.0002*	0.0002*	0.0002*	0.0002*	0.0002*	0.0002*	0.0002*	0.0002*	0.0002*	0.0002*	0.0003*	0.0002*	0.0002*	0.0002*	0.0002*	0.0002*	0.0002*	0.0002*
CTR versus D14	0.0006*	0.0002*	0.0006*	0.0003*	0.0002*	0.0002*	0.0002*	0.0002*	0.0002*	0.0002*	0.0006*	0.0002*	0.0006*	0.0002*	0.0002*	0.0002*	0.0002*	0.0002*	0.0002*	0.0002*
D2 versus D7	0.0002*	0.0002*	0.0002*	0.0002*	0.0002*	0.0002*	0.9591	0.0379	0.0002*	0.9591	0.5054	0.5054	0.7209	0.7209	0.6454	0.5054	0.0002*	0.5737	0.0207	0.0499
D2 versus D14	0.0002*	0.0002*	0.0002*	0.0002*	0.0002*	0.0002*	0.0002*	0.0207	0.0002*	0.3823	0.6454	0.0047	0.9591	0.5054	0.1605	0.2345	0.0002*	0.0148	0.0019	0.0003*
D7 versus D14	0.1605	0.3282	0.0002*	0.3823	0.8785	0.5737	0.0002*	0.6454	0.0002*	0.6454	0.7209	0.1304	0.5737	0.1304	0.1049	0.083	0.8785	0.1304	0.7984	0.0019

Abbreviations: AB, alcian blue; CD31, platelet endothelial cell adhesion molecule; CD34, vascular endothelium marker; DESMIN, muscle‐specific intermediate filament desmin; CLDN, claudin; CK7, cytokeratin 7; CK14, cytokeratin 14; DSP, desmoplakin; FHUS, full‐thickness human urethra substitute; PSR, picrosirius red; SMA‐ACT, α‐smooth muscle actin; SMOO, smoothelin; TJP1, tight junction protein 1; UPK1b, uroplakin 1b; VCAN, versican.

* *p* Values below 0.001.

**FIGURE 4:**
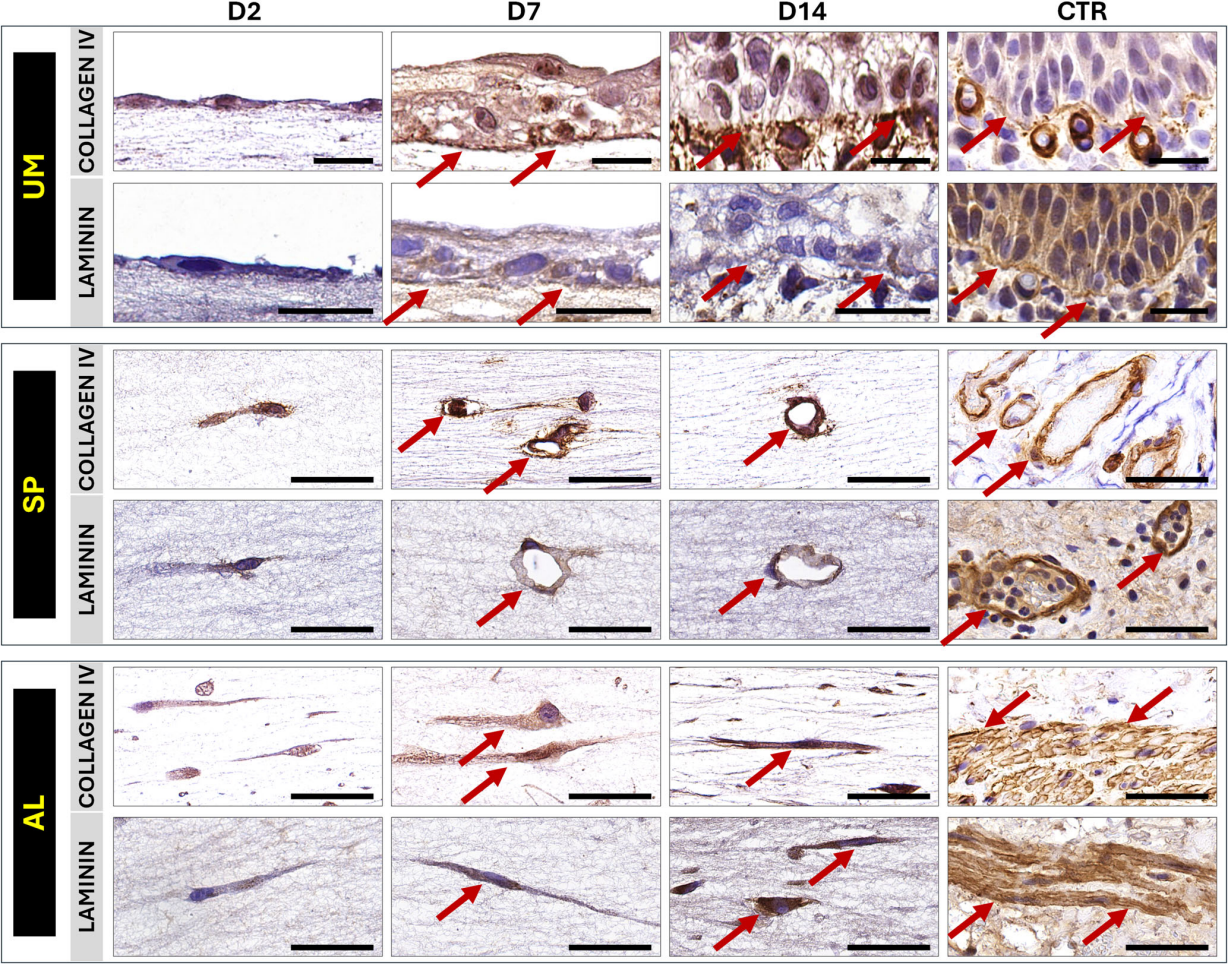
Immunohistochemical analysis of basement membrane components (collagen IV and laminin) in each specific bioartificial tissue layer generated in this work (urethral mucosa layer [UM], spongy layer [SP], and tunica albuginea layer [AL]) kept in culture for 2 days (D2), 7 days (D7), and 14 days (D14), and in the native human urethra used as control (CTR). Illustrative areas showing positive signal are highlighted with arrows. Images correspond to illustrative pictures obtained from each type of sample, with a sample size of *n* = 8 per group. Scale bars: 20 μm.

Then, we analyzed two key components of the basement membrane in UM (Figure 4 and Table 2), and we found that bioartificial UM tissues kept in culture for 2 days were negative for collagen IV and laminin, whereas CTR was strongly positive for both markers. Bioengineered UM samples were mildly positive for collagen IV and slightly positive for laminin at Days 7 and 14.

**TABLE 2:** Semiquantitative expression analysis of basement membrane markers collagen IV and laminin in each layer of the human urethra substitute generated by tissue engineering (urethral mucosa layer [UM], spongy layer [SP], and tunica albuginea layer [AL]) and kept in culture for 2 days (D2), 7 days (D7), and 14 days (D14) and in the human native urethra used as control (CTR) (*n* = 8). The immunohistochemical signal was assessed as strong (+++), mild (++), slight (+), or negative (−).

	UM		SP		AL	
	Collagen IV	Laminin	Collagen IV	Laminin	Collagen IV	Laminin
D2	−	−	++	++	−	−
D7	++	+	+++	++	+	−
D14	++	+	+++	++	+++	++
CTR	+++	+++	+++	+++	+++	+++

**FIGURE 5:**
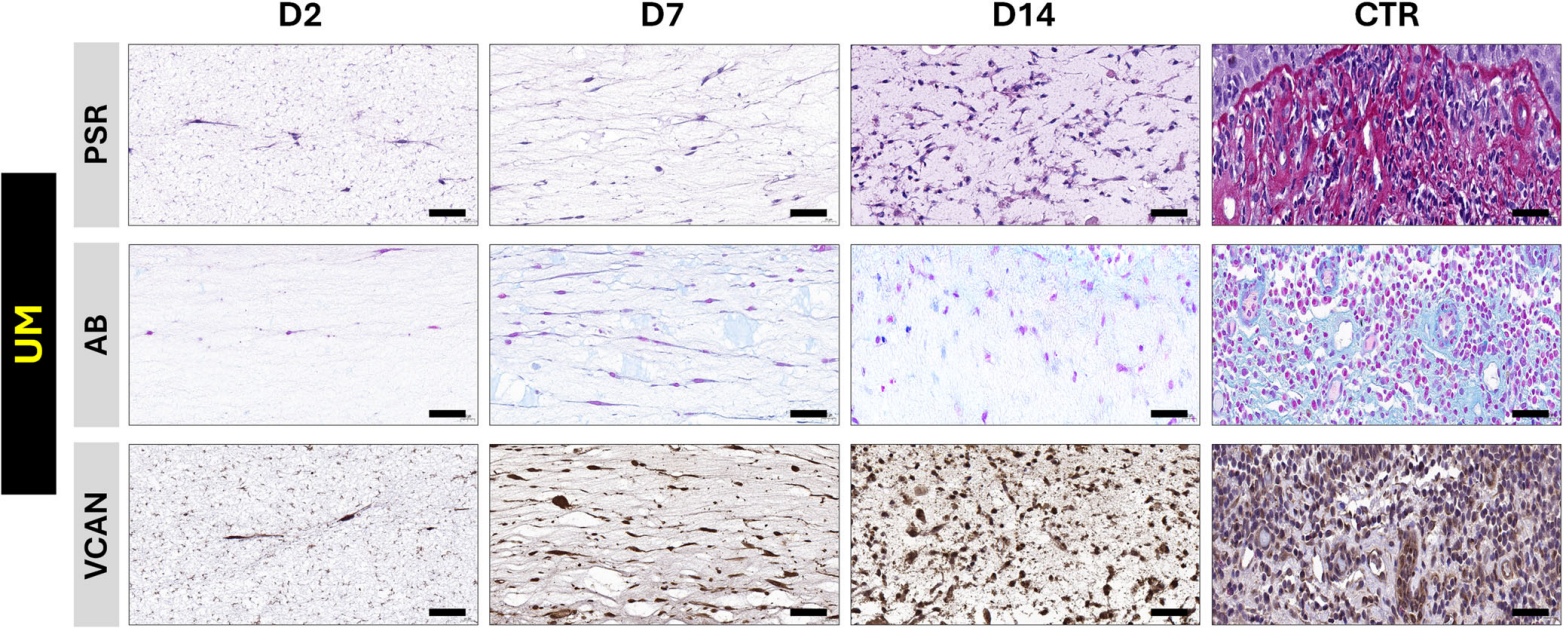
Analysis of stromal extracellular matrix components in the bioengineered urethral mucosa layer (UM) generated by tissue engineering and kept in culture for 2 days (D2), 7 days (D7), and 14 days (D14), and in the native human urethra used as control (CTR) using histochemistry and immunohistochemistry. AB, alcian blue; PSR, picrosirius red; VCAN, versican. Images correspond to illustrative pictures obtained from each type of sample, with a sample size of *n* = 8 per group. Scale bars: 50 μm.

On the other hand, evaluation of ECM components in the stromal layer of the UM revealed highly positive signal for PSR, AB, and versican in CTR samples, with statistically significant differences among all samples (Figure 5 and Table 1). For PSR, we found that artificial UM samples corresponding to 2 and 7 days of development showed low levels of PSR signal, with a significant increase at Day 14, although these tissues were significantly lower than CTR tissues. For AB, all bioengineered UM tissues were significantly lower than CTR, with no differences among samples at Days 2, 7, and 14. For versican, the lowest levels corresponded to Day 2 samples, with a significant increase at Day 7, and a significant increase at Day 14, although all bioartificial samples were significantly lower than CTR.

### 3.4 Histochemical and immunohistochemical analysis of the spongy layer generated by tissue engineering and CTR tissues

In the case of the SP layer, we first found that CTR tissues were strongly positive for the basement membrane components collagen IV and laminin, whose expression was restricted to the areas surrounding the abundant blood vessels found at this level (Figure 4 and Table 2). On Day 2 of development, bioengineered SP samples were mildly positive in the cells scattered throughout the biomaterial. Then, samples at Days 7 and 14 showed strong collagen IV signal around the blood vessels, as it was the case for the CTR samples, whereas these bioengineered tissues remained mildly positive for laminin at Days 7 and 14, at lower levels than CTR.

**FIGURE 6:**
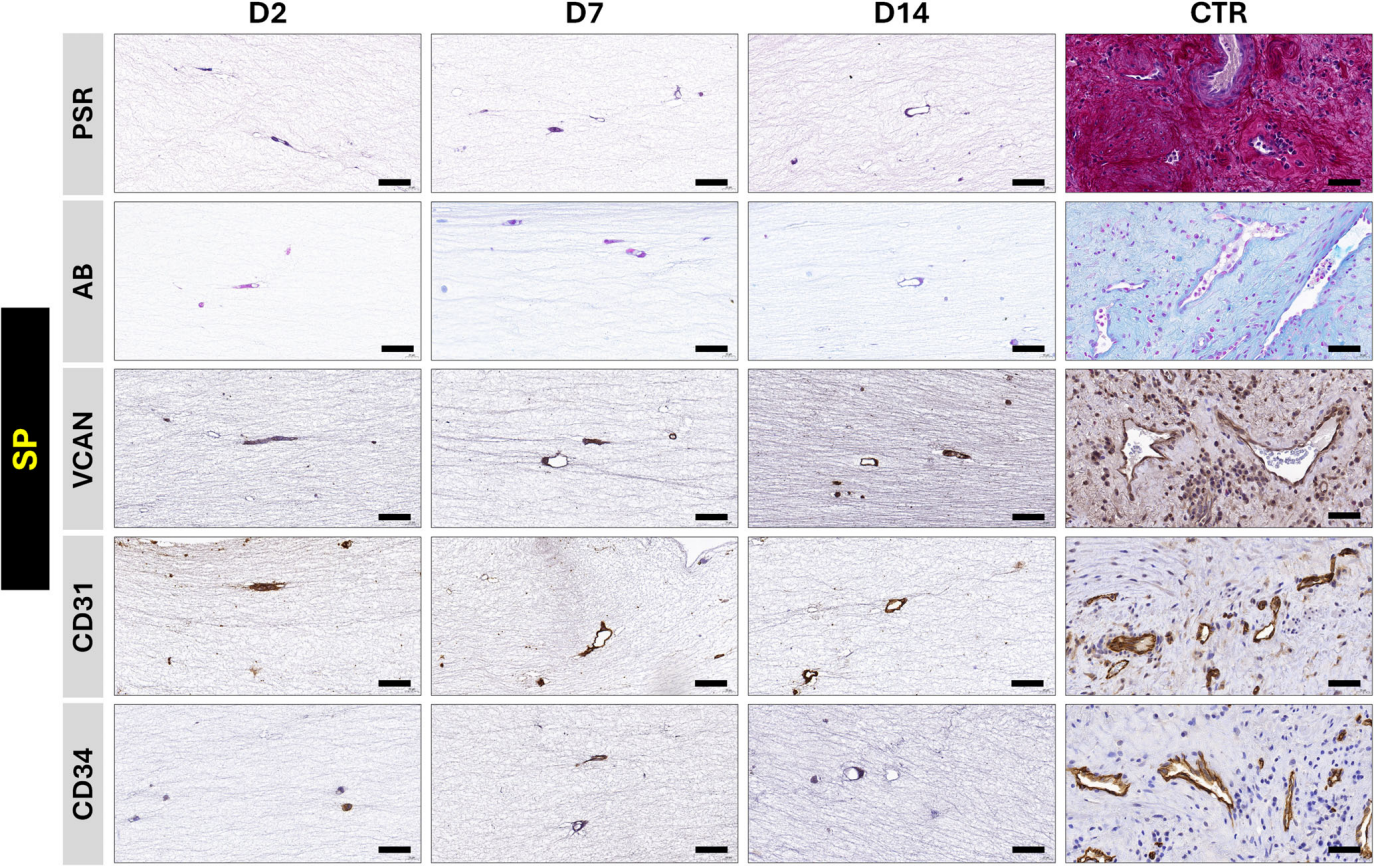
Analysis of stromal extracellular matrix components and vascular components in the bioengineered spongy layer (SP) generated by tissue engineering and kept in culture for 2 days (D2), 7 days (D7), and 14 days (D14), and in the native human urethra used as control (CTR) using histochemistry and immunohistochemistry. AB, alcian blue; CD31, platelet endothelial cell adhesion molecule; CD34, vascular endothelium marker CD34; PSR, picrosirius red; VCAN, versican. Images correspond to illustrative pictures obtained from each type of sample, with a sample size of *n* = 8 per group. Scale bars: 50 μm.

In addition, our analysis of key components of the tissue ECM using PSR and AB histochemistry and versican immunohistochemistry revealed very low signal in bioartificial SP tissues kept in culture for 2, 7, and 14 days, and very high signal in CTR tissues, with differences between native and artificial tissues being statistically significant (Figure 6 and Table 1). Finally, we evaluated the presence of blood vessels using CD31 and CD34 immunohistochemistry. As for the ECM components, we found non‐significant differences among bioengineered SP tissues corresponding to 2, 7, and 14 days of follow‐up, whereas differences were significant with CTR tissues (Figure 6 and Table 1).

### 3.5 Histochemical and immunohistochemical analysis of the tunica albuginea layer generated by tissue engineering and CTR tissues

Analysis of key components of the basement membrane in the AL layer of the native urethra (CTR samples) showed a strongly positive signal restricted to muscle cells allocated at this layer (Figure 4 and Table 2). For collagen IV, we found that bioartificial AL was negative at Day 2, slightly positive at Day 7, and strongly positive at Day 14 of follow‐up. For laminin, our results showed a negative signal in bioartificial AL at Days 2 and 7, and a mildly positive signal at Day 14 of development.

**FIGURE 7:**
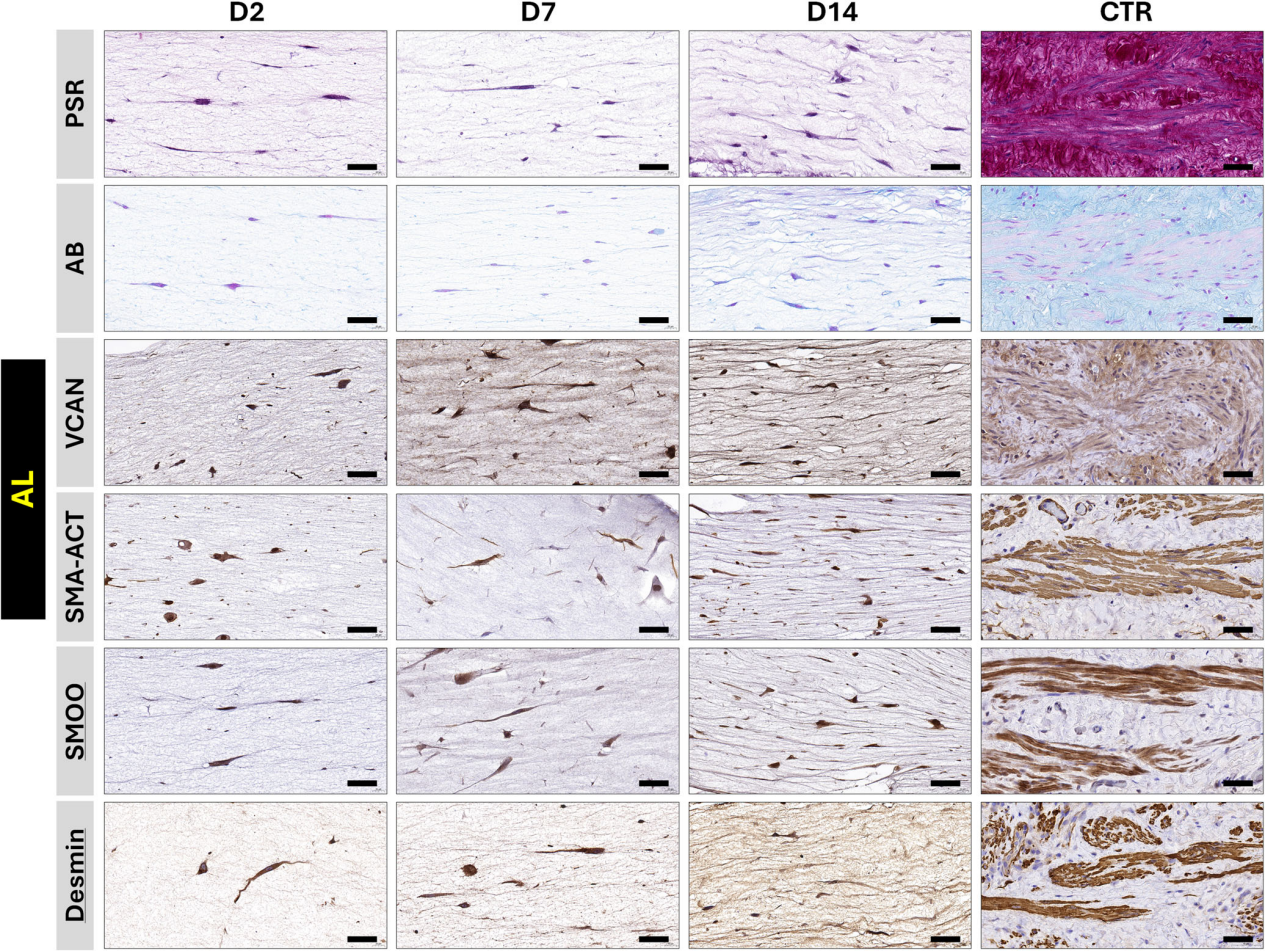
Analysis of stromal extracellular matrix components and smooth muscle components in the bioengineered tunica albuginea layer (AL) generated by tissue engineering and kept in culture for 2 days (D2), 7 days (D7), and 14 days (D14), and in the native human urethra used as control (CTR) using histochemistry and immunohistochemistry. AB, alcian blue; Desmin, muscle‐specific intermediate filament desmin; PSR, picrosirius red; SMA‐ACT, α‐smooth muscle actin; SMOO, smoothelin; VCAN, versican. Images correspond to illustrative pictures obtained from each type of sample, with a sample size of *n* = 8 per group. Scale bars: 50 μm.

When ECM components were analyzed in the AL layer of the native CTR tissues, we found that the strongest histochemical and immunohistochemical signal corresponded to CTR, which contained significantly higher collagen fibers, proteoglycans, and versican, as determined by PSR, AB, and versican immunohistochemistry, respectively, than bioengineered AL (Figure 7 and Table 1). For PSR and AB, bioengineered tissues showed low staining signal, with non‐significant differences among culture times. However, versican immunohistochemistry showed a significant increase from Day 2 to Day 7, although Day 7 and Day 14 samples were not statistically different.

Furthermore, the immunohistochemical analysis of three proteins associated with smooth muscle cell differentiation showed that the native CTR tissues expressed significantly higher levels of SMA‐ACT, SMOO, and desmin than bioengineered AL (Figure 7 and Table 1). For SMA‐ACT and SMOO, bioengineered tissues corresponding to different follow‐up times showed non‐significant differences. However, we found a trend to increase with time the expression of desmin in bioengineered AL, with statistically significant differences between Day 2 and Day 14 samples for this immunohistochemical marker (Figure 7 and Table 1).

### 3.6 Generation of a full‐thickness substitute by tissue engineering

**FIGURE 8:**
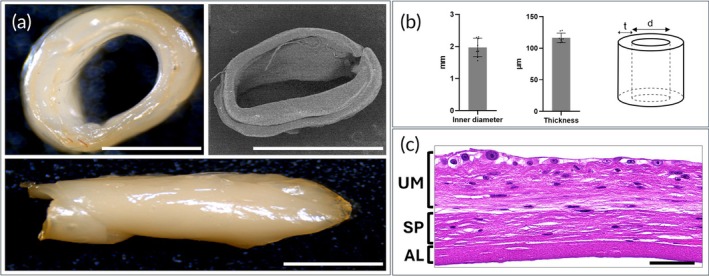
Full‐thickness human urethra substitute (FHUS) generated by tissue engineering by combining the urethral mucosa layer (UM), spongy layer (SP), and tunica albuginea layer (AL) layers kept in culture for 14 days. Panel (a): gross aspect of the bioartificial tissue in a transversal plane (macroscopic view and low‐magnification scanning electron microscopy image), and a longitudinal plane. Panel (b): quantitative measurements of the diameter (*d*) and thickness (*t*) of the tubular FHUS structures generated by tissue engineering. Panel (c): histological analysis of the complete FHUS stained with hematoxylin and eosin (HE), showing the three layers that make up this structure, including the UM, the SP, and the AL. Scale bars: 2 mm in (a), and 50 μm in (c).

Once the individual layers were characterized, we combined the UM, SP, and AL corresponding to 14 days of development in a single structure to generate a FHUS (Figure 8). The macroscopic analysis of this FHUS showed an elongated tubular structure with an internal lumen (Figure 8a) of 1.96 ± 0.89 mm in diameter and 116.50 ± 7.41 μm thick (Figure 8b). When this structure was analyzed histologically using HE staining (Figure 8c), we found that the FHUS consisted of a single structure in which several layers of dense material containing different types of cells could be detected. A superficial epithelium was found at the top of the FHUS, which was overlying a dense material containing elongated cells corresponding to the UM. Below this mucosa, we found two layers of fibrillar material containing cells, with the most apical layer, corresponding to the SP, showing abundant spaces among the fibers of the material, and the most profound layer, corresponding to the AL, showing densely packed biomaterial fibers.

### 3.7 Analysis of biomechanical properties of bioengineered tissues and control porcine urethras

**FIGURE 9:**
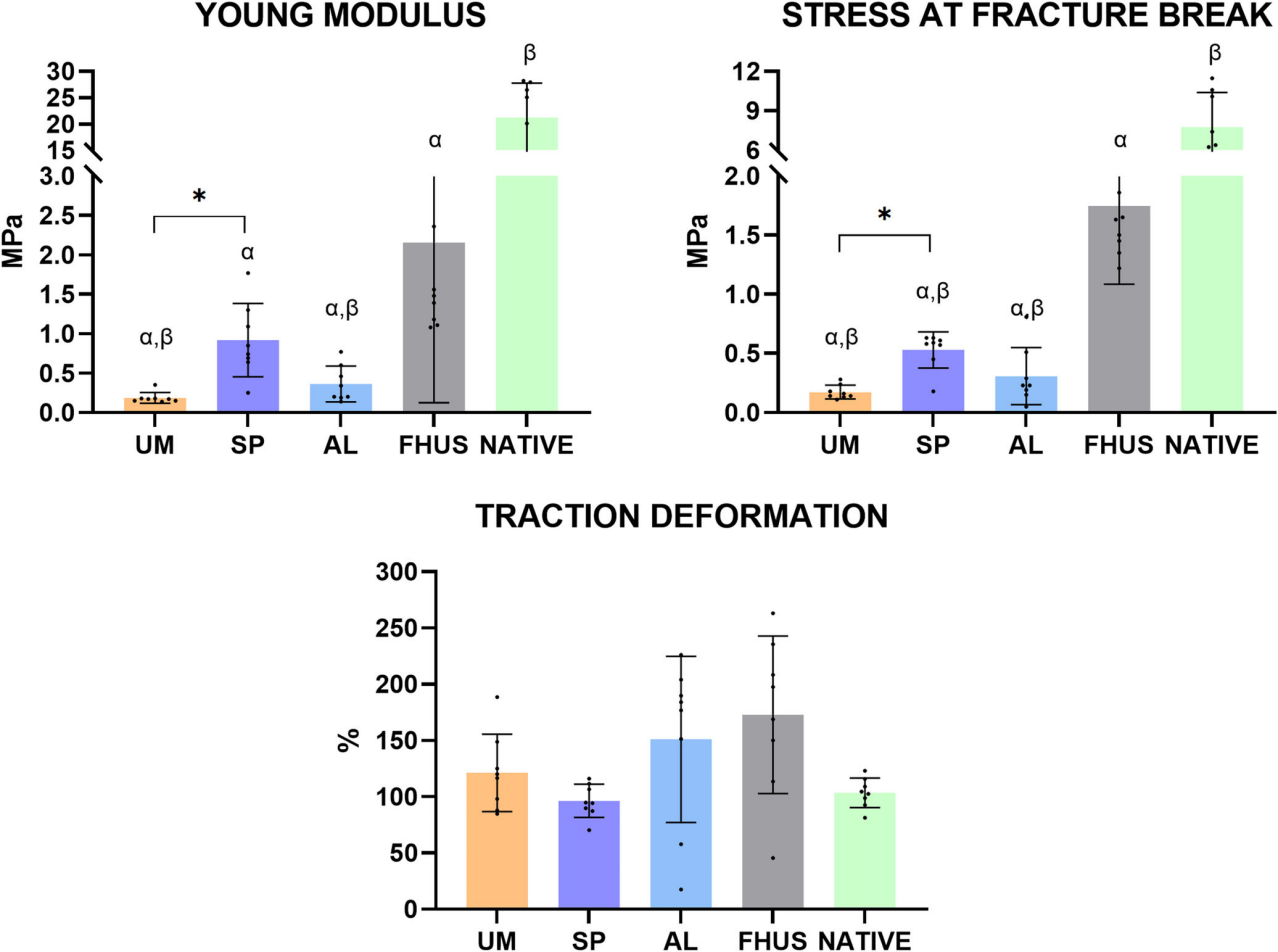
Biomechanical properties of the bioengineered tissues generated in this study and kept in culture for 14 days, along with the control native porcine urethra (*n* = 8 per group). Bar graphs display individual experimental replicates (dots) and error bars for each biomechanical property. Statistical comparisons between two specific groups of samples were performed using the Mann‐Whitney test. Statistical *p* values below 0.001 are labeled with alpha (*α*) for significant differences versus the native control, beta (*β*) for differences versus FHUS, and asterisks (*) for differences between two specific layers (urethral mucosa layer [UM], spongy layer [SP], and tunica albuginea layer [AL]). FHUS, full‐thickness human urethra substitute; NATIVE, native porcine urethra used as control.

An analysis of the biomechanical behavior of the different tissue substitutes generated in this work (UM, SP, AL, and FHUS) and control porcine urethras revealed that, in general, all samples were more elastic than stiff, as the traction deformation was higher than the Young modulus (Figure 9). First, we found that the Young modulus was low in UM, SP, and AL samples, and the generation of a FHUS resulted in a significant increase in this parameter as compared with UM and AL. However, none of the bioengineered tissues reached the levels found in the native urethra, whose Young modulus was higher than that of the rest of the samples. A similar behavior was found for the stress at fracture break, with a significant increase in FHUS as compared with UM, SP, and AL samples, although levels were significantly lower than those found in native tissues. However, the analysis of the traction deformation revealed non‐significant differences among samples, with all tissue types showing a high degree of traction deformation (above 100% in most cases).

## 4 DISCUSSION

Several models of bioartificial substitutes of the human urethra have been generated by tissue engineering.^13^, ^17^, ^31^ However, most of these substitutes are acellular or contain only one type of cells, and full‐thickness urethral substitutes containing the three main layers of this organ have not been reported to date. In this regard, it has been suggested that tissues generated by tissue engineering should be able to biomimetically reproduce the complex structure of multilayer organs, which results in a significant improvement in the usefulness of these structures.^32^

Different biofabrication methods have been described for the development of multilayered tissues, including 3D bioprinting,^33^ combination with different types of membranes^34^ or additive biofabrication methods,^35^ among others. In addition, we have previously described a biofabrication method allowing the combination and fusion of several layers of bioengineered tissues in a single structure, that was applied to the generation of a full‐thickness substitute of the rabbit hard palate.^18^ This method was based on plastic compression nanostructuration protocols previously applied to improve the biomechanical properties of biomaterials used in tissue engineering of the human cornea,^36^ skin,^11^ nerve^37^ and other structures. In agreement with these previous reports, we demonstrated that the application of nanostructuration to tissue engineering of the human urethra can be efficiently used to generate a FHUS containing the three main layers of this organ.

In the present work, we first generated bioengineered substitutes of each layer of the human urethra, including the UM, SP, and AL layers, and we found that cells were highly viable within the fibrin or fibrin‐agarose biomaterials used to generate each tissue layer. These results are in agreement with previous reports from our group demonstrating that these biomaterials are highly biocompatible, and cells cultured in these materials retain high levels of cell viability,^22^ which could contribute to explaining the good results obtained when fibrin‐agarose bioartificial tissues were grafted in patients.^11^, ^12^ In addition, the analysis of cell proliferation showed that bioartificial tissues generated in the laboratory tend to actively proliferate during the first days of development, but tend to reduce proliferation as the tissues acquire higher differentiation profiles. This phenomenon is not surprising, since it has been demonstrated that proliferation and differentiation may be antagonistic processes that tend to behave in opposite ways.^38^

A thorough preclinical validation and characterization of bioengineered tissues generated in the laboratory is a crucial requirement for future clinical translation.^39^ For that reason, each bioengineered layer (UM, SP, and AL) was characterized to determine the main histological, histochemical, and immunohistochemical features of these structures, before using them to generate a FHUS. When the bioengineered UM was evaluated and characterized, we found that this structure was partially biomimetic to the native UM regarding the presence of a stratified epithelium and a subjacent stromal layer, as it is the case of the native UM.^2^ When the epithelial layer of the bioengineered UM was analyzed, we found that this structure tended to evolve with time in culture, with a progressive stratification of the epithelial layer from Day 2 to Day 7, although cells were not able to reach the differentiation levels found in the native epithelium, as previously suggested for other bioengineered tissues kept in culture.^18^, ^40^ A proper differentiation and organization of the epithelial layer is an important requirement of artificial tissues, since it has been demonstrated that the biological function of this epithelium is strictly dependent on cell organization and differentiation.^41^ In this regard, our analysis of relevant epithelial cell markers at the epithelial layer of bioartificial UM showed a progressive increase in the expression of all the analyzed markers, including several cytokeratins, cell–cell junctions, and the specific marker of urothelial cells UPK1b. Although the high expression levels found in CTR tissues were not reached after the follow‐up time, our results suggest that bioartificial UM were partially differentiated and shared important molecular similarities with CTR tissues, despite terminal differentiation not being reached during the analyzed follow‐up times. Similar results were found for the development of a basement membrane below the epithelial layer, with the sequential development of collagen IV and laminin, without reaching the levels found in CTR tissues. Again, it is well known that the development of a complex structure like the basement membrane depends on the presence of numerous induction factors generated at the stromal and the epithelial layers, and full differentiation is seldom achieved ex vivo.^42^ When the stromal layer of the UM was analyzed, we found a progressive time‐dependent synthesis of relevant components of the stromal ECM, although the expression levels found in CTR were not reached in culture. In general, bioengineered UM stroma showed a significant increase in collagen fibers and in versican molecules with culture time, without reaching the amounts of the CTR tissues. In agreement with the results found for the epithelial and basement membrane layers of the bioengineered UM, these findings suggest that UM tissues kept in culture tend to progressively mature and differentiate, but a complete biomimesis to the CTR was not achieved ex vivo. Similar results were reported for several types of bioartificial tissues kept in culture, such as the human cornea, oral mucosa, and skin.^36^, ^40^, ^43^

Evaluation of the SP layer generated by tissue engineering showed a regular material in which the endothelial cells cultured within were able to form hollow structures that were morphologically similar to capillary vessels from Day 7 of follow‐up. Endothelial cells were previously reported to have the potential to form small blood vessels when cultured on different biomaterials,^44^ including fibrin.^45^ As for the UM, characterization of the SP layer showed that the bioengineered SP contained very low amounts of collagen fibers, proteoglycans, and versican, compatible with an undifferentiated tissue substitute, and remained stable with the culture time. However, the microvessels generated within this structure showed positive expression of the vascular differentiation markers CD31 and CD34^24^ and the basement membrane components collagen IV and laminin, indicating that these capillaries displayed a certain level of differentiation within the biomaterial, although the number of blood vessels remained invariable after 2, 7, and 14 days of development in the bioengineered SP. In general, these findings support the generation of a SP substitute in the laboratory using fibrin biomaterials, despite this tissue being poorly differentiated regarding the ECM components. The fact that microvessels were present in this tissue and remained stable with the culture time upholds the use of the bioengineered SP as a biological substitute for a well‐vascularized structure like the SP. For future clinical use, however, SP substitutes containing higher amounts of microvessels, along with other cell types able to improve ECM differentiation, should be developed by tissue engineering.

Finally, we analyzed and characterized the AL substitute generated in this work, and we found that the leiomyocytes cultured within fibrin‐agarose biomaterials were able to grow and reached partial differentiation. Although these cells were not able to form the well‐organized fibrillar bundles found in CTR tissues, the cells showed positive expression of basement membrane components that typically surround muscle cells in native tissues,^46^ as well as the relevant markers of smooth muscle cells SMA‐ACT, SMOO, and desmin.^25^ In contrast, and in agreement with the results found for the SP substitute, the bioengineered AL expressed low amounts of the ECM components collagen, proteoglycans, and versican, as compared to CTR tissues. Again, these results are compatible with a partial differentiation of the AL tissues generated by tissue engineering. Smooth muscle cells have been previously cultured on different types of biomaterials for use in tissue engineering of the urine bladder, among other tissues.^47^ However, this is the first time that these cells are cultured within fibrin‐agarose biomaterials.

Once each individual layer was characterized, we combined UM, SP, and AL layers to generate a single multilayered FHUS structure. Interestingly, our biomechanical assessment of each individual layer of tissue revealed that these layers were very elastic, although their biomechanical behavior was very distant from that of the native urethra. However, the generation of a FHUS contributed to improving the biomechanical properties of this urethral substitute, with significant differences with the single layers generated in the laboratory, especially for the stress at fracture break, and with the UM and AL for the Young modulus, although the stiffness and resistance of the native urethra were not reached. However, the biomechanical parameters related to the deformation properties of the different tissues (traction deformation) were comparable to those of the native urethra. Though future works should be carried out to improve the biomechanical resistance of these bioartificial organs using crosslinking or other methods,^48^ the present work demonstrated that the generation of a FHUS using nanostructuration methods contributed to improving the biomechanical properties of monolayered tissues. However, it is important to note that we used the male porcine urethra as a control for the biomechanical analyses. While the native porcine urethra shares high similarities to the human urethra, including the same histological structure, consisting of UM, SP, and AL layers, some differences may exist, particularly in the thickness of each layer.^49^, ^50^ Additionally, it is well known that the properties of the male urethra may significantly differ from those of the female urethra^2^ and we used only the male porcine urethra as a control. These limitations of the present study should be taken into account to interpret the results of the biomechanical studies, and extrapolation of these results to the human urethra, especially to the female urethra, must be done with caution. Future studies should be carried out to analyze the biomechanical behavior of the native male and female human urethras.

In addition to the biomechanical analyses, we evaluated the FHUS at the histological level, and we found that the nanostructuration methods described in the present work were able to generate a full‐thickness multilayered substitute by assembling the UM, SP, and AL layers in a single structure. Although the cell viability, histochemical, and immunohistochemical characterization were carried out on the individual layers instead of the full‐thickness substitute, previous studies carried out by our research group demonstrated that the nanostructuration process does not significantly alter cell viability or tissue composition,^18^ and we might hypothesize that the structure and composition of each specific layer found in the FHUS (UM, SP, and AL) are the same as those found in the individual layers kept in culture. Future studies should analyze the FHUS to confirm the histological, histochemical, and immunohistochemical properties of the full‐thickness organ substitutes generated by tissue engineering, as well as the viability of cells found at each layer of this FHUS. Another limitation of the present work is the lack of studies evaluating the long‐term stability of the FHUS in terms of tissue architecture, cell viability, and molecular identity. Although the individual layers demonstrated favorable levels of cell survival, partial differentiation, and structural integrity in culture up to 14 days, these findings cannot be fully extrapolated to the multilayered tissue, and future studies will be required to assess the structural organization, integration, and functionality of the entire FHUS over time.

In summary, in this work we have been able to generate a human urethra substitute showing partial biomimetism to the human urethra, not only at the structural level, with the three main layers of this organ (UM, SP, and AL), but also at the molecular level, with expression of key components by each layer. The use of nanostructuration enabled the generation of a multilayered structure, allowing the generation of a tubular structure. Although in vivo studies should demonstrate the potential of this tissue substitute as a bioartificial urethra, the positive results obtained in this work suggest that this FHUS could have potential usefulness in the tissue engineering of the human urethra.

## AUTHOR CONTRIBUTIONS

**David Sánchez‐Porras**: Data curation, formal analysis, investigation, methodology, validation, visualization, writing—original draft, writing—review and editing. **Miguel Etayo‐Escanilla**: Data curation, investigation, methodology, validation, writing—review and editing. **José‐Andrés Moreno‐Delgado**: Investigation, methodology. **María del Mar Lozano‐Martí**: Investigation, methodology. **Fabiola Bermejo‐Casares**: Investigation, methodology. **Miguel Alaminos**: Conceptualization, data curation, formal analysis, methodology, project administration, supervision, visualization, writing—original draft, writing—review and editing. **Jesús Chato‐Astrain**: Conceptualization, formal analysis, investigation, supervision, validation, writing—review and editing. **Fernando Campos**: Conceptualization, formal analysis, investigation, supervision, validation, writing—review and editing. **M. Carmen Sánchez‐Quevedo**: Conceptualization, funding acquisition, project administration, resources, supervision, writing—original draft, writing—review and editing. **Ricardo Fernández‐Valadés**: Conceptualization, funding acquisition, project administration, resources, supervision, writing—original draft, writing—review and editing.

## FUNDING INFORMATION

This work was supported by the Spanish “Plan Estatal de Investigación Científica, Desarrollo e Innovación Tecnológica” (I + D + i) of the Spanish Ministry of Science, Innovation and Universities (Instituto de Salud Carlos III), Grant FIS PI22/00059. Cofinanced by the European Regional Development Fund (FEDER/ERDF) through the “*Una manera de hacer Europa*” program, European Union.

## CONFLICT OF INTEREST STATEMENT

None of the authors have a conflict of interest to disclose.

## Supporting information


**Table S1.** Antibodies and technical conditions for the immunohistochemical analyses carried out in this work.

## Data Availability

The data set generated in this study is openly available in the public repository Zenodo at https://doi.org/10.5281/zenodo.14510547, with the license Creative Commons Attribution 4.0 International.
