# Supplementary material for: Generation and ex vivo characterization of a full‐thickness substitute of the human urethra by tissue engineering
Source: Bioeng Transl Med. 2025 Sep 16;11(3):e70049. doi: 10.1002/btm2.70049 (PMC13247409; doi:10.1002/btm2.70049)
Supplement: Supplementary file 1 — Table S1. Antibodies and technical conditions for the immunohistochemical analyses carried out in this work. [file BTM2-11-e70049-s001.docx]

| **Antibody** | **Dilution/Incubation** | **Pretreatment** | **Reference** |
| --- | --- | --- | --- |
| Rabbit monoclonal  anti-KI67 (SP6) | Prediluted  Overnight at 4 ˚C | EDTA buffer pH 8  20 min at 95 ˚C | *Master Diagnóstica* (Granada, Spain) *ref: MAD-000310QD* |
| Rabbit polyclonal anti-versican | 1:100  Overnight at 4 ˚C | EDTA buffer pH 8  20 min at 95 ˚C | Abcam (Bristol, England) ref: ab19345 |
| Mouse monoclonal  anti-collagen type IV | Prediluted  Overnight at 4 ˚C | EDTA buffer pH 8  20 min at 95 ˚C | *Master Diagnóstica* (Granada, Spain) *ref: MAD-001060QD* |
| Rabbit polyclonal  anti-laminin | 1:250  Overnight at 4 ˚C | Citrate buffer pH 6  20 min at 95 ˚C | Abcam (Bristol, England) ref: ab11575 |
| Mouse monoclonal anti-cytokeratin 14 (LL002) | Prediluted  Overnight at 4 ˚C | EDTA buffer pH 8  20 min at 95 ˚C | *Master Diagnóstica* (Granada, Spain) ref: MAD-005103QD |
| Mouse monoclonal anti-cytokeratin 7 (OV-TL 12/30) | Prediluted  Overnight at 4 ˚C | Citrate buffer pH 6  20 min at 95 ˚C | *Master Diagnóstica* (Granada, Spain) *ref: MAD-001004QD* |
| Rabbit monoclonal Anti-Uroplakin Ib (UPKIB/8976R) | 1:100  Overnight at 4 ˚C | Citrate buffer pH 6  20 min at 95 ˚C | Novus Biologicals (Cambridge, England) ref: NBP3-23743 |
| Mouse monoclonal anti-CD31 Antibody (JC/70A) | Prediluted  Overnight at 4 ˚C | EDTA buffer pH 8  20 min at 95 ˚C | *Master Diagnóstica* (Spain) *ref:* MAD-002048QD |
| Mouse monoclonal anti-CD34 (Clone QB-End/10) | Prediluted  Overnight at 4 ˚C | Citrate buffer pH 6  20 min at 95 ˚C | *Master Diagnóstica* (Spain) *ref:*  MAD-001613QD |
| Mouse monoclonal anti-SMA-ACT (1A4) | Prediluted  Overnight at 4 ˚C | Citrate buffer pH 6  20 min at 95 ˚C | *Master Diagnóstica* (Spain) *ref: MAD-001195QD* |
| Mouse monoclonal anti-smoothelin (R4A) | Prediluted  Overnight at 4 ˚C | EDTA buffer pH 8  20 min at 95 ˚C | *Master Diagnóstica* (Spain) *ref:* MAD-000445QD |
| Rabbit monoclonal anti-desmin (Y66) | 1:75  Overnight at 4 ˚C | EDTA buffer pH 8  20 min at 95 ˚C | Abcam (England) ref: ab32362 |
| Mouse monoclonal anti-desmoplakin 1-2 (DPI/II 236.23.1) | Ready-to-use  1 hour at RT | Citrate buffer pH 6  20 min at 95 ˚C | Origene () ref: AM09122SU-N |
| Rabbit polyclonal Anti-TJP1 | 1:50  Overnight at 4 ˚C | EDTA buffer pH 8  20 min at 95 ˚C | Merck (Germany) ref:  HPA001637 |
| Rabbit polyclonal anti-claudin-1 | Prediluted  Overnight at 4 ˚C | Citrate buffer pH 6  20 min at 95 ˚C | *Master Diagnóstica* (Spain) *ref:* MAD-000523QD |
| ImmPRESS® HRPAnti-Mouse IgG (Peroxidase) | 1h at RT | - | Vector Laboratories (EEUU) (ref: MP-7401) |
| ImmPRESS® HRP Anti-Rabbit IgG (Peroxidase) | 1h at RT | - | Vector Laboratories EEUU (ref: MP-7402) |
| ImmPRESS® HRP  Anti-Goat IgG (Peroxidase) | 1h at RT | - | Vector Laboratories (EEUU) (ref: MP-7405) |
| Goat IgG anti-mouse (whole molecule)-FITC antibody | - | - | Merck (Germany) ref: F0257 |
| Goat IgG anti-rabbit (whole molecule)-FITC antibody | - | - | Merck (Germany) ref: F9887 |

**Suppementary Table S1**. Antibodies and technical conditions for the immunohistochemical analyses carried out in this work.
